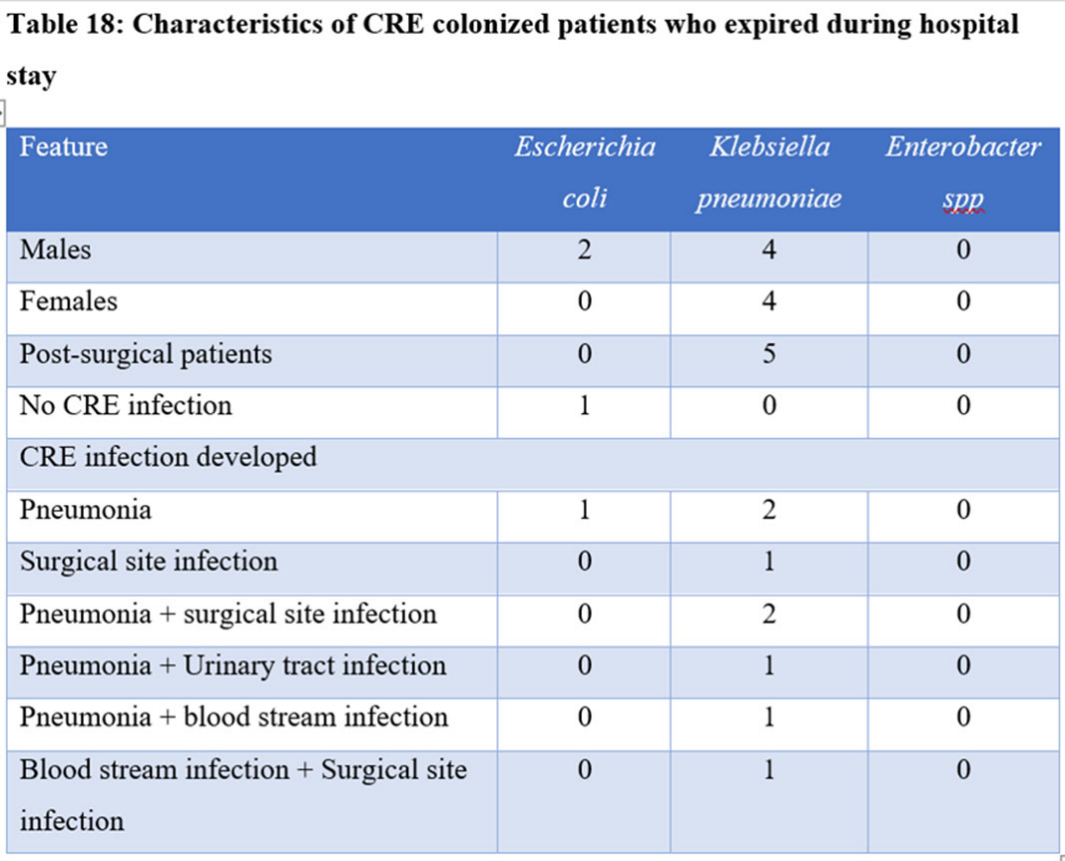# An Observational Study on CRE Colonization and Subsequent Risk of Infection in Adult ICU Patients

**DOI:** 10.1017/ash.2021.130

**Published:** 2021-07-29

**Authors:** Kirtika Sharma, Vibhor Tak, Vijaya Lakshmi Nag

## Abstract

**Background:** Carbapenem-resistant Enterobactericeae (CRE) has emerged as a global health threat with increasing incidence. It is a particular problem in India because control over antibiotics prescription is really poor; these agents can be easily bought over the counter and the antibiotic prescription threshold is low among Indian doctors. Also, even when administered, antibiotics are given in inappropriate dosages and durations. CRE infections are a healthcare challenge due to their difficulty to treat and high morbidity and mortality. Colonization requires infection prevention measures, and it should be prioritized. **Methods:** We sought to determine the prevalence rate of CRE colonization in the gastrointestinal tract in newly admitted ICU patients along with follow-up of any subsequent infection following colonization. A prospective observational study was carried out among ICU patients from January 2019 to August 2020 by collecting perirectal swabs from patients who gave consent. Clinical variables were identified, and the relationship between CRE colonization and subsequent systemic CRE infection was assessed. Processing was carried out by culturing on MacConkey agar plate with ertapenem disk and further identified using conventional microbiological techniques. The ertapenem MIC was determined using an Epsillometer (E) test. The modified carbapenem inactivation (mCIM) test and the EDTA carbapenem inactivation method (eCIM) were used to confirm carbapenem resistance using Clinical Laboratory Standards Institute 2020 guidelines (Figure 1). **Results:** Among 192 ICU patients, 37 (19.27%) were colonized with CRE (Table 1). Also, 13 (35.13%) CRE isolates showed metallo-β-lactamase resistance. Furthermore, 18 CRE isolates (48.64%) showed serine carbapenemase activity; 6 CRE isolates showed no carbapenemase activity. *Klebsiella pneumoniae* (n = 25 of 37, 67.56%) was the most common CRE isolated followed by *Escherichia coli* (n = 11 of 37, 29.72%) and 1 isolate of *Enterobacter* spp (n = 1 of 37, 0.02%). Of 37 patients, 33 (89.18%) developed CRE infection during their hospital stay. Pneumonia was the most common infection developed (36.36%), followed by surgical site infection (21.21%) and urinary tract infection (12.12%). Only 1 patient developed a bloodstream infection. However, 9 patients (27.27%) developed multiple-site infections. Of 37 CRE-colonized patients, 10 (27.02%) died during their hospital stay. **Conclusions:** Our study highlights the increased risk of CRE infection and mortality in patients with CRE colonization in ICU patients. Hence, CRE perirectal screening for detection of asymptomatic carriers should be conducted, and strict infection control measures, such as isolation and cohorting with barrier nursing of such patients, should be done to prevent further spread of CREs in hospital settings.

**Funding:** No

**Disclosures:** None

**Figure 1.**
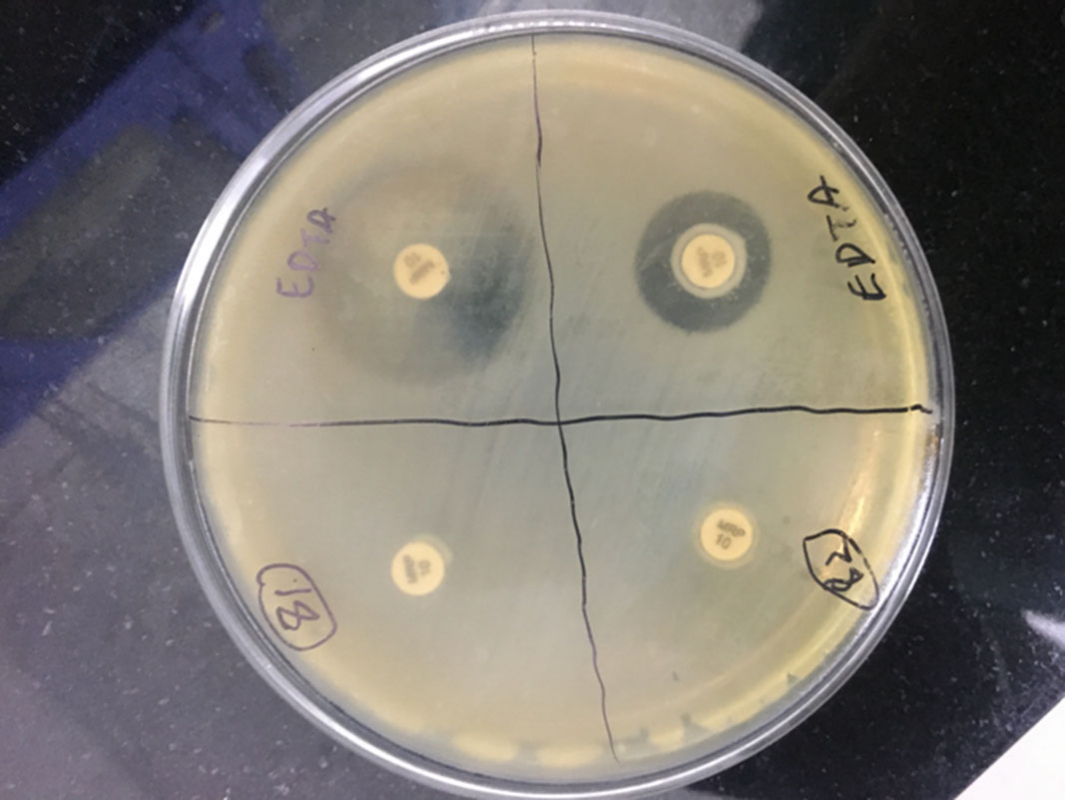

**Table 1.**
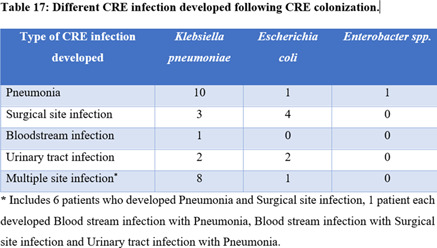

**Table 2.**